# Destruction, disruption and disaster: Sudan’s health system amidst armed conflict

**DOI:** 10.1186/s13031-023-00542-9

**Published:** 2023-09-27

**Authors:** Alaa Dafallah, Osman K. O. Elmahi, Maisoon Elbukhari Ibrahim, Rania Elfatih Elsheikh, Karl Blanchet

**Affiliations:** 1 Centre for Tropical Medicine and Global Health, Nuffield Department of Medicine, University of Oxford, Peter Medawar Building for Pathogen Research, South Parks Road, Oxford, OX1 3SY UK; 2 Faculty of Medicine, University of Khartoum, Khartoum, Sudan; 3 Faculty of Medicine, Ibn Sina University, Khartoum, Sudan; 4 Institute of Global Health, Faculty of Medicine, University of Geneva, Geneva, Switzerland; 5 Faculty of Dentistry, University of Khartoum, Khartoum, Sudan; 6 Geneva Centre of Humanitarian Studies, Faculty of Medicine, University of Geneva, Geneva, Switzerland

**Keywords:** Sudan, Armed conflict, Health system, Humanitarian crisis, Violence against health workers, Attacks on healthcare, Health system resilience

## Abstract

The ongoing armed conflict in Sudan has resulted in a deepening humanitarian crisis with significant implications for the country's health system, threatening its collapse. This article examines the destruction, disruption, and disastrous consequences inflicted upon Sudan's health system. The conflict has led to the severe compromise of healthcare facilities, with only one-third of hospitals in conflict zones operational. Artillery attacks, forced militarization, power outages, and shortages of medical supplies and personnel have further crippled the health system. The exodus of health workers and escalating violence have exacerbated the crisis. Disrupted service delivery has resulted in the interruption of essential health services, including obstetric care, emergency services, and dialysis. Financial losses to the health system are estimated at $700 million, impacting an already underfunded sector. We identify that in addition to restoration of peace and mobilization of urgent aid, immediate prioritization of the reconstruction of the health system is crucial to mitigate the long-term consequences of the war. Rebuilding a resilient health system is sine qua non for Sudan's progress towards universal health.

Sudan is spiraling into a deep humanitarian crisis following the eruption of armed clashes on April 15th between the Sudanese Armed Forces (SAF) and the Rapid Support Forces (RSF) in the capital city of Khartoum, North Kordofan, Darfur and River Nile states. According to the most recent report from the Ministry of Health on the 9th of May, 2023, 550 civilians were killed, 4926 injured and at least one million displaced [1]. Beyond the direct costs of war on civilians, the inconspicuous side of reality is the indirect and long-term impact this war has and will have on the health system.

## Destruction of health infrastructure

Healthcare services have been severely compromised. As of 23rd July, less than one third of hospitals in conflict zones are functional, with 70% of hospitals out of service [2]. Of the 59 hospitals out of service in conflict zones, 17 were attacked by artillery and 20 were evacuated, of which 12 have been forcibly militarized and converted into barracks by the RSF. The remaining hospitals suspended services due to power outages, shortage of fuel for generators, lack of medical supplies and critical lack of health workers. Additionally, the RSF also seized multiple public health assets critical for service delivery including the National Public Health Laboratory, the Central Blood Bank, and the National Medical Supplies Fund, contributing to critically low medical supplies and blood reserves across several other states [3]. The siege of the National Public Health Laboratory constitutes a biological hazard, increasing the risk of multiple outbreaks of polio, measles, and cholera due to insecure containment [4].

In-service hospitals are reporting severe health worker shortages [2]. Health workers are among thousands that have fled the capital since the start of the war severely limiting capacity in hospitals. The remaining health workers are either unable to access health facilities due to fear for their safety or are exhausted, burdened by acute shortages in specialized cadres such as surgeons and anesthetists and medical supplies.

Violence against health workers, albeit not new to Sudan, has escalated [5]. Since the commencement of the conflict, 13 health workers were killed, 4 have been abducted by militia and 9 are reported missing [3]. “I have to hide my stethoscope when I am going to see a sick person in the neighbourhood,” expressed a young doctor in Khartoum on the 7th of May, “otherwise the militia groups may abduct me to take care of their wounded. It’s sad that we are a target”.

The Sudanese Doctors Syndicate has condemned the attacks on health workers as a violation of the Geneva Convention and Additional Protocol II, in line with several calls by the UN, WHO and professional associations late April [6, 7]. However, the absence of accountability mechanisms amidst the collapse of state institutions has limited the enforcement of existing national law.

## Disruption of service delivery

The weakness of the country's monitoring and health information systems impedes the accurate estimation of the impact of disrupted service delivery resulting from the war. Anecdotal evidence collected by the authors points to the disruption of key life-saving health services, obstetric and newborn care, emergency care for trauma or medical emergencies, dialysis, cancer care etc. [8, 9]. Dire calls were made by the National Center for Kidney Diseases and Surgery (NCKDS) on the 27th of April, following the bombing and evacuation of Alshaheeda Salma Kidney Centre, which offered free dialysis sessions [1, 10]. “We need doses of immunosuppressants and 140,000 dialyses filters as soon as possible. The situation is scary, and we may lose 12,000 human beings soon,” urged the Director of the Centre, Dr Nazar Zulfu.

Similarly, access to health services has been severely interrupted for the 968,000 Sudanese refugees crossing borders to Egypt, Ethiopia, South Sudan, Chad, Central African Republic and Libya [11]. More than 3,200,000 people are newly internally displaced across Sudan, overwhelming the health systems in respective states. However, despite efforts to provide health services for displaced persons and refugees, the need for health services remains disproportionately higher.

## Disastrous consequences

The already frail health system in Sudan is on the brink of collapse. This has detrimental consequences on the response to ongoing outbreaks of dengue fever. Equally, there is an increased risk of emerging outbreaks such as cholera due to worsening water sanitation and hygiene conditions further aggravated by the upcoming flood season [12]. Therefore continued and heightened disease surveillance is necessary to overcome the risk of outbreaks and epidemics [13]. Furthermore, in the absence of catch-up campaigns, the disruption to immunization services increases the threat of outbreaks of vaccine-preventable diseases such as measles, risking the reversal of progress made over the past decade in under-5 mortality, maternal mortality, and incidence of communicable diseases.

The financial losses of the health system have been estimated at $700 m [3]. This will heavily impact the public health sector, which in addition to being chronically underfunded, is experiencing acute constraints subsequent to a 1.4% GDP contraction in 2023 and the mobilization of funds to military and defense functions [14]. Without rapid restoration of peace and proper investments on the reconstruction of the health system, this war will have long-term consequences on progress towards universal health coverage and global health security.

As the war completes its 100-day mark, the situation in Sudan is to say the least, disastrous. The international and humanitarian response to Sudan’s crisis has not only been slow, but strikingly disparate to responses in recent crises such as Ukraine. We call for immediate ceasefire, the creation of conduits for humanitarian access and the mobilization of urgent funds, lifesaving aid and essential medicines. Provision of free health services to fleeing refugees and vulnerable displaced persons must be availed by host states. Above all, rebuilding a resilient health system in Sudan is an immediate priority and should be planned for during these turbulent times to mitigate the long-term consequences of this war. Therefore, we call on the international community of experts and researchers to convene, interrogate, derive evidence, and support the reconstruction and strengthening of what is now, a failing health system in Sudan.
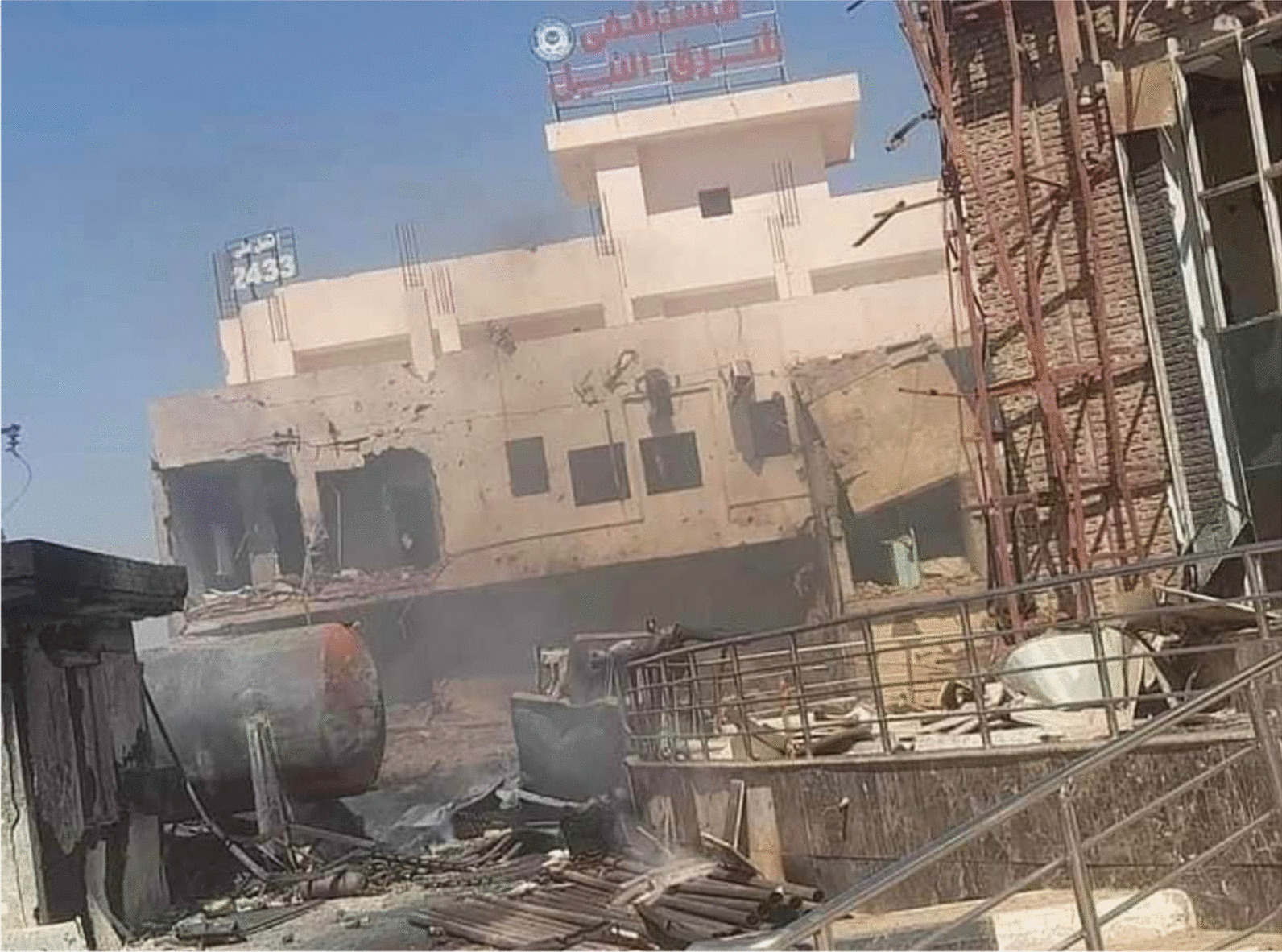


Image shows the damage inflicted by an artillery attack on Sharg El Neel (East Nile) Hospital on the 15th of May 2023. This hospital has been out of service for close to a month, following its evacuation and militarization late April. Source: Mustafa Emad Maghazi [15].

## Data Availability

Not applicable.
